# Crystal structure of a tetra­nuclear copper(II) complex with 1,10-phenanthroline and 3-nitro­phthalate ligands

**DOI:** 10.1107/S2056989026005116

**Published:** 2026-05-19

**Authors:** Naima Karimova, Khayit Turaev, Gulvar Muqumova, Jabbor Suyunov, Maftuna Primkulova, Lidiya Izotova

**Affiliations:** a Termez State University, A Navoiy Str, 43, Termez, 190100, Uzbekistan; b Denau Institute of Entrepreneurship and Pedagogy, Bog Str, 112, Denau, 733500, Uzbekistan; c Institute of Bioorganic Chemistry, Academy of Sciences of Uzbekistan, H. Abdullaev Str,83, Tashkent, 100125, Uzbekistan; University of Aberdeen, United Kingdom

**Keywords:** crystal structure, tetra­nuclear copper(II) complex, 1,10-phenanthroline, 3-nitro­phthalate

## Abstract

In the hydrated complex tetra­kis­(μ-2-carb­oxy-6-nitro­benzoato-κ^2^*O*:*O*′)bis­(μ-3-nitro­benzene-1,2-di­carboxyl­ato-κ^2^*O*:*O*′)tetra­kis­[(1,10-phenanthroline-κ*N*:*N*′)copper(II)] hexahydrate, [Cu_4_(C_8_H_4_NO_6_)_4_(C_8_H_3_NO_6_)_2_(C_12_H_8_N_2_)_4_]·6H_2_O, the Cu^II^ centres exhibit distorted coordination geometries defined by nitro­gen atoms from chelating 1,10-phenanthroline ligands and oxygen atoms from 3-nitro­phthalate anions. The centrosymmetric mol­ecular assembly is consolidated by bridging carboxyl­ate groups, while the crystal packing is governed by hydrogen bonding and π–π stacking inter­actions between aromatic rings. The scattering contribution of one disordered water mol­ecule contribution was treated using the SQUEEZE procedure.

## Abstract 2

A tetra­nuclear copper(II) complex assembled from 1,10-phenanthroline and 3-nitro­phthalate ligands features carboxyl­ate-bridged metal centres and supra­molecular stabilization *via* hydrogen bonding and aromatic π–π stacking inter­actions.

## 1. Chemical context

Aromatic di­imine ligands such as 1,10-phenanthroline are widely employed in coordination chemistry owing to their strong chelating ability, rigid planar geometry, and pronounced π-acceptor character (Constable, 1987 ▸). Copper(II) complexes incorporating phenanthroline frequently exhibit diverse structural motifs and supra­molecular behaviour. Polycarboxyl­ate ligands derived from 3-nitro­phthalic acid represent versatile building units capable of multiple coordination modes. The presence of carboxyl­ate and nitro functionalities enables structural diversity and promotes the formation of multinuclear assemblies (Thompson, 2002 ▸).
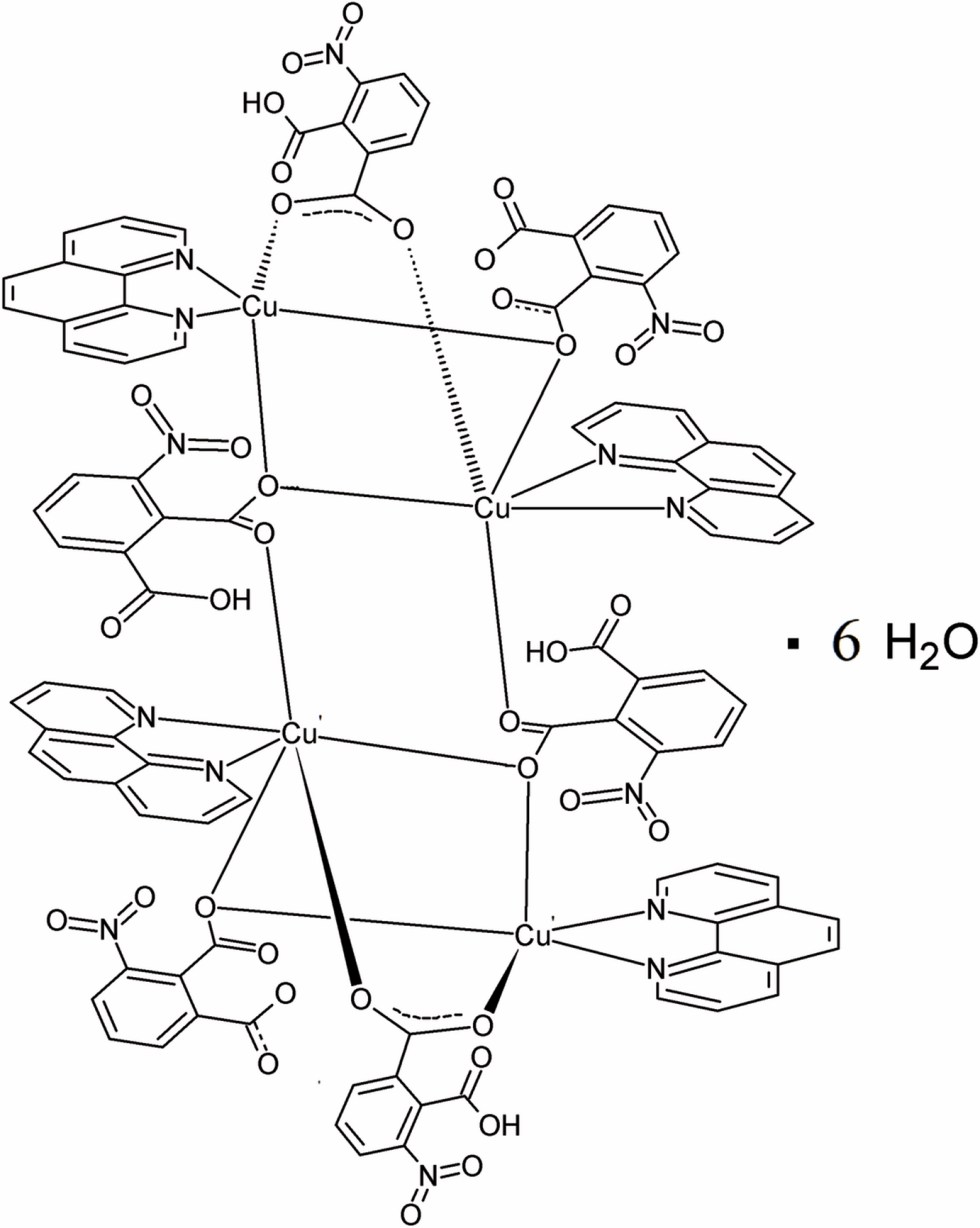


In the present work, we report how the combination of Cu^II^, 1,10-phenanthroline (C_12_H_8_N_2_) and 3-nitro­phthalic acid (C_8_H_4_NO_6_) in the mixed solvents of *N*,*N*-di­methyl­formamide, ethanol and water results in the title tetra­nuclear copper complex, [Cu_4_(C_8_H_4_NO_6_)_4_(C_8_H_3_NO_6_)_2_(C_12_H_8_N_2_)_4_]·6H_2_O (**I**).

## 2. Structural commentary

**Figure 1:**
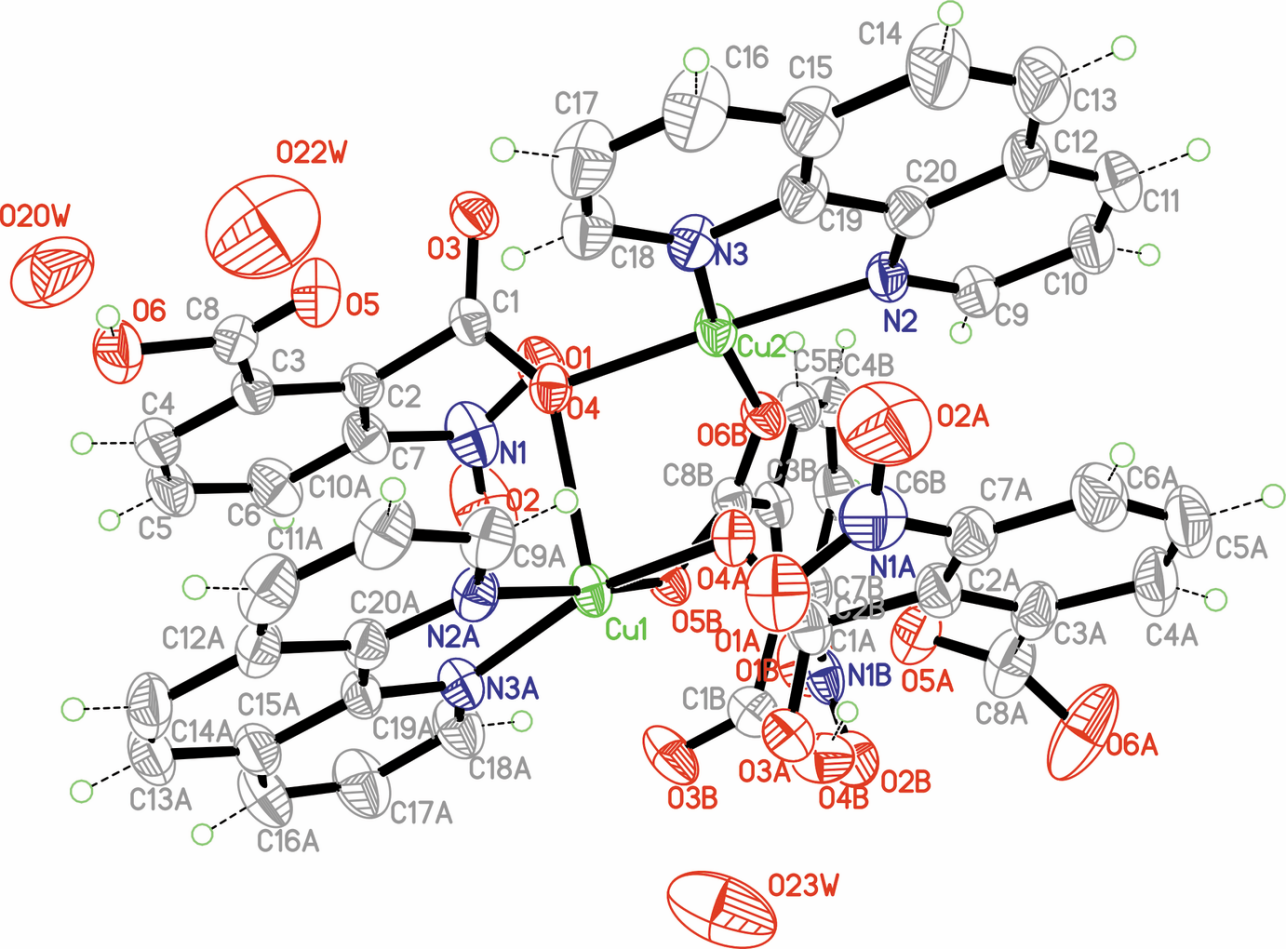
The mol­ecular structure of the asymmetric unit of (**I**) with displacement ellipsoids drawn at the 30% probability level.

The structure of (**I**) consists of a centrosymmetric, tetra­nuclear, copper(II) grouping in which the metal centres are inter­connected by 3-nitro­phthalate ligands and further ligated by chelating 1,10-phenanthroline donors. Two crystallographically independent copper atoms are present in the asymmetric unit (Fig. 1 ▸).

Atom Cu1 is five-coordinate and adopts a distorted square-pyramidal geometry. The basal plane is defined by two nitro­gen atoms from one chelating phenanthroline ligand and two oxygen atoms from carboxyl­ate groups (Table 1 ▸). The apical position is occupied by atom O4: the pronounced elongation of this axial Cu—O bond relative to the equatorial distances is consistent with the expected Jahn–Teller distortion of a 3*d*^9^ Cu^II^ centre.

Atom Cu2 is four-coordinate within the asymmetric unit and exhibits a distorted square-planar geometry defined by two nitro­gen atoms from phenanthroline and two oxygen atoms from carboxyl­ate groups. These Cu—O and Cu—N bond lengths fall within the typical ranges observed for Cu^II^ complexes containing carboxyl­ate and di­imine ligands.

The 3-nitro­phthalate ligand acts as a μ_2_-bridging linker connecting adjacent copper(II) centres. Atom O4 functions as an asymmetric carboxyl­ate bridge between Cu1 and Cu2, coordinating axially to Cu1 [Cu1—O4 = 2.314 (3) Å] and equatorially to Cu2 [Cu2—O4 = 1.972 (3) Å]. The significant difference in bond lengths indicates stronger equatorial coordination to Cu2 and a weaker axial inter­action with Cu1, consistent with Jahn–Teller distortion commonly observed for Cu^II^ centres.

**Figure 2:**
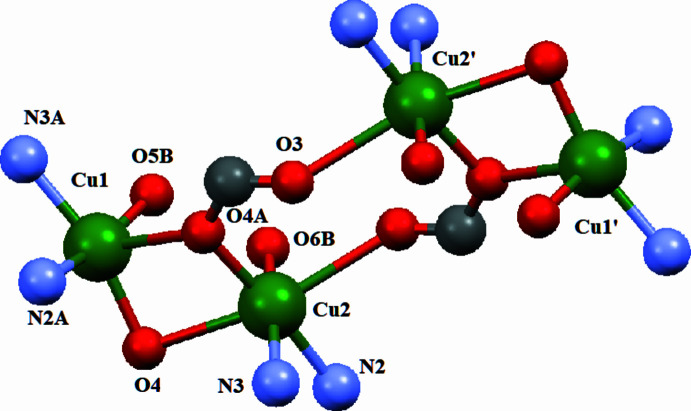
Simplified representation of the tetra­nuclear Cu_4_ core in (**I**) showing the butterfly-type arrangement and the μ_2_-carboxyl­ate bridges. Primed atoms are generated by the symmetry operation 1 − *x*, 1 − *y*, 1 − *z*.

Additional carboxyl­ate oxygen atoms, O5*B* and O6*B*, coordinate to Cu1 and Cu2, respectively, further consolidating the tetra­nuclear Cu_4_ core through μ_2_-carboxyl­ate bridges (Fig. 2 ▸).

The C8*A*—O5*A* and C8*A*—O6*A* bond lengths [1.261 (7) and 1.256 (8) Å, respectively] are nearly identical, indicating delocalization within the carboxyl­ate group and confirming its deprotonated state. Accordingly, this nitro­phthalate ligand is presumed to be present in a doubly deprotonated form.

Taking into account the presence of two such dianionic ligands together with four monodeprotonated nitro­phthalate ligands and four Cu^II^ centres, the overall charge of the complex is balanced.

## 3. Supra­molecular features

In the crystal, aromatic π–π stacking inter­actions are observed between symmetry-related aromatic rings. The centroid–centroid separation is 3.687 (3) Å for the *Cg*1⋯*Cg*2^i^ inter­action [symmetry code: (i) 1 − *x*, 1 − *y*, 1 − *z*; C*g*1 is the centroid of the N2/C9–C12/C20 pyridyl ring of the phenanthroline ligand and *Cg*2 is the centroid of the C2–C7 benzene ring of the nitro­phthalate ligand].

The inter­planar separation is 3.665 Å, with a dihedral angle of 4.0 (3)°, indicating that the rings are nearly parallel. The very small slippage (ca 0.4 Å) suggests an almost face-to-face arrangement of the inter­acting aromatic systems (Moulton & Zaworotko, 2001 ▸).

**Figure 3:**
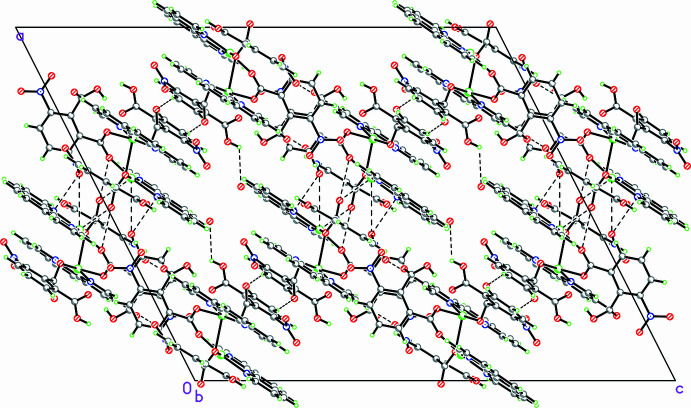
Crystal packing of (**I**) viewed along the [010] direction.

O—H⋯O and C—H⋯O hydrogen bonds (Table 2 ▸) are also present. The packing is showin in Fig. 3 ▸.

## 4. Hirshfeld surface analysis

Hirshfeld surface analysis was performed using *CrystalExplorer* (Turner *et al.*, 2017 ▸; Spackman *et al.*, 2021 ▸) to qu­antify the inter­molecular inter­actions in the crystal structure.

**Figure 4:**
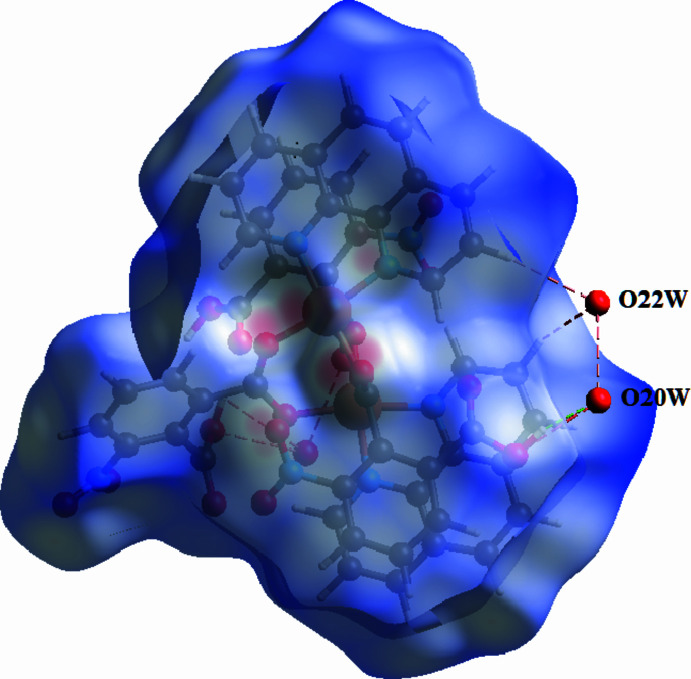
The Hirshfeld surface of (**I**) mapped over *d*_norm_ showing short inter­molecular O⋯H/H⋯O contacts as red regions.

**Figure 5:**
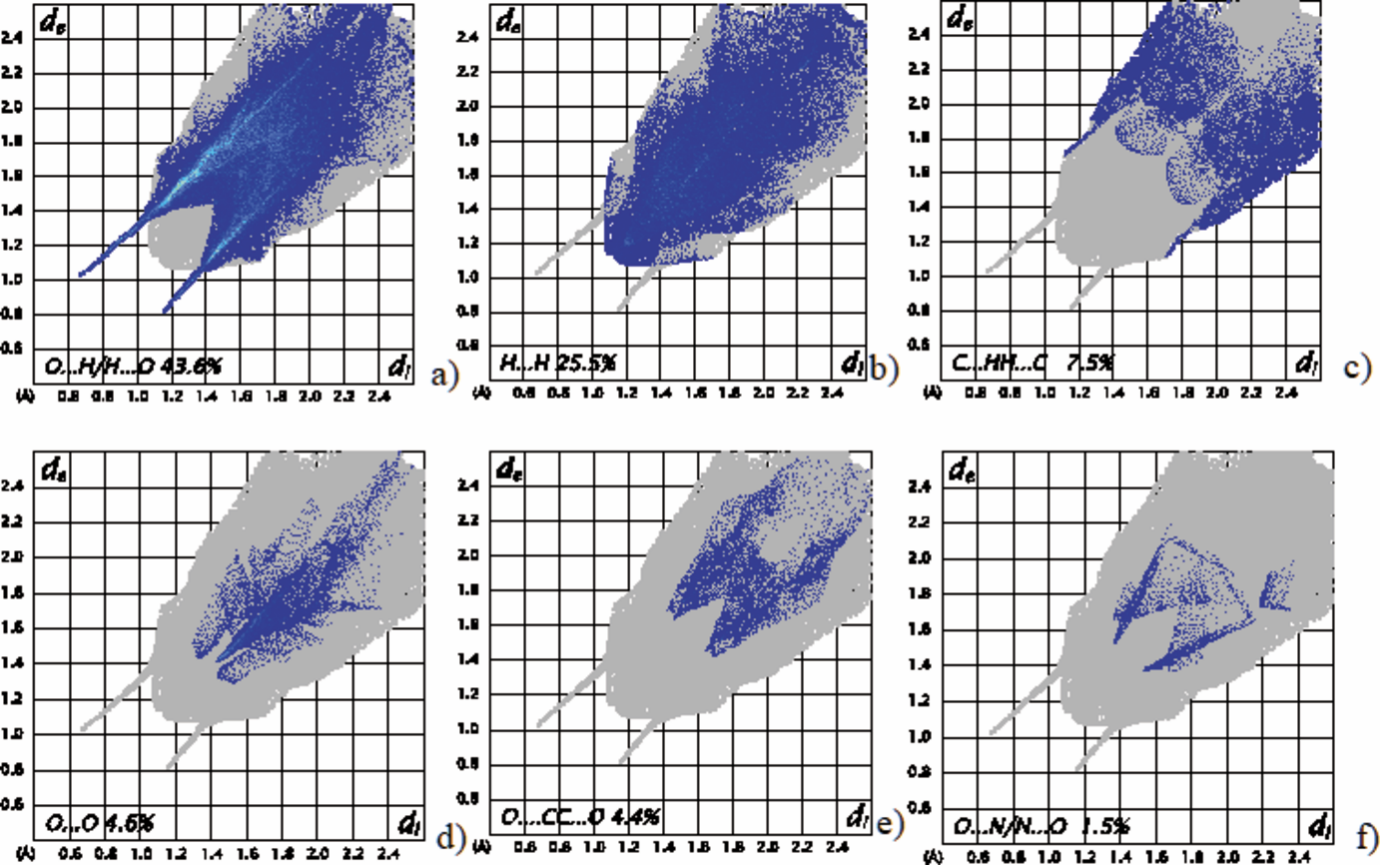
The two-dimensional fingerprint plots for (**I**) showing the percentage contributions of (*a*) O⋯H/H⋯O, (*b*) H⋯H, (*c*) C⋯H/H⋯C, (*d*) O⋯O, (*e*) O⋯C/C⋯O and (*f*) O⋯N/N⋯O contacts.

The Hirshfeld surface mapped over *d*_norm_ displays prominent red regions corresponding to short O⋯H/H⋯O contacts, confirming the dominant role of hydrogen bonding in consolidate the crystal packing. (Fig. 4 ▸). Two-dimensional fingerprint plots (Fig. 5 ▸) show that O⋯H/H⋯O contacts contribute 43.6%, followed by H⋯H inter­actions (25.5%). C⋯H/H⋯C contacts account for 7.5%, while O⋯O (4.6%), O⋯C/C⋯O (4.4%) and O⋯N/N⋯O (1.5%) inter­actions provide minor contributions. These results confirm that hydrogen bonding and dispersion inter­actions dominate the supra­molecular architecture.

## 5. SQUEEZE treatment

Examination of the difference-Fourier map revealed regions of diffuse residual electron density located within solvent-accessible voids, consistent with the presence of highly disordered solvent mol­ecules. Attempts to model this electron density using discrete atomic positions resulted in unstable refinements. Accordingly, the solvent contribution was treated using the SQUEEZE (Spek, 2015 ▸) procedure implemented in *PLATON* (Spek, 2020 ▸). The procedure identified solvent-accessible voids with volumes of approximately 10–48 Å^3^, containing up to 13 electrons per void and resulted in stable refinement behaviour and chemically reasonable structural parameters. The reported mol­ecular formula, density, *etc*. refer only to the ordered portion of the structure.

## 6. Database survey

A search of the Cambridge Structural Database (CSD Version 2025.3.1; Groom *et al.*, 2016 ▸) for structures containing both ‘1,10-phenanthroline’ and ‘Cu^2+^’ yielded 359 hits, highlighting the widespread use of phenanthroline as a chelating ligand in copper(II) coordination chemistry.

A more specific search combining the terms ‘phenanthroline + Cu2^+^ + benzene­carb­oxy­lic acid’ returned ten entries (CSD refcodes ETTIE, BOVCAT, CAMZIA, CODVOJ, DOGDVB, HOPYAM, TEJXEL, XEHTEL, RADYIF and CABCIV). Among these, the structures with refcodes RADYIF (Zhu *et al.*, 2004 ▸) and CABCIV (Pinto *et al.*, 2020 ▸) represent the structurally closest known analogues to the present compound.

RADYIF features a ladder-like tetra­nuclear Cu_4_ core sustained by μ_2_- and μ_3_-carboxyl­ate bridges in combination with chelating 1,10-phenanthroline ligands. Similarly, the stepped tetra­nuclear core in CABCIV exhibits mixed square-planar and square-pyramidal coordination geometries around the Cu^II^ centres, consistent with the Jahn–Teller distortion typically observed for 3*d*^9^ metal ions.

Despite these structural similarities, the title compound is distinguished by the presence of six 3-nitro­phthalate ligands per Cu_4_ unit, resulting in an increased degree of μ_2_-carboxyl­ate connectivity and a more extensively bridged metal framework. In contrast to RADYIF and CABCIV, the present structure displays a higher ligand-to-metal bridging ratio, leading to a more compact tetra­nuclear core. Furthermore, the supra­molecular architecture in (**I**) is reinforced by O—H⋯O hydrogen-bonding inter­actions and pronounced slipped π–π stacking between adjacent phenanthroline ligands, which contribute significantly to the packing. These combined structural features differentiate the title compound from the closest CSD analogues and highlight its enhanced connectivity and packing consolidation.

## 7. Synthesis and crystallization

3-Nitro­phthalic acid (1.00 mmol, 0.211 g) was dissolved in *N*,*N*-di­methyl­formamide (DMF), 1,10-phenanthroline (1.00 mmol, 0.180 g) was dissolved in ethanol, and Cu(CH_3_COO)_2_ (1.00 mmol, 0.18 g) in distilled water. The mixture of solutions of 3-nitro­phthalic acid and copper(II) acetate were combined in a flat-bottom flask and stirred for 20 min using a magnetic stirrer. The solution of 1,10-phenanthroline was added dropwise. The reaction mixture was stirred at 333 ± 0.5 K for an additional 20 min.

The resulting solution was left to stand at room temperature in a loosely covered vessel, maintaining a pH of approximately 6.0. After 12 days, blue prism-shaped crystals of (**I**) suitable for X-ray diffraction formed at the bottom of the vessel. The crystals were isolated by filtration.

## 8. Refinement

Crystal data, data collection and structure refinement details are summarized in Table 3 ▸. Hydrogen atoms were placed geometrically and refined using a riding model. One solvent-accessible region containing highly disordered electron density, probably corresponding to water mol­ecule(s) of crystallization, could not be modelled satisfactorily. The contribution of this diffuse solvent was treated using the SQUEEZE procedure as implemented in *PLATON* (Spek, 2020 ▸). Three additional water mol­ecules were located from difference-Fourier maps. However, their hydrogen atoms could not be positioned reliably due to unfavorable geometry and large displacement parameters. These hydrogen atoms were therefore omitted from the refinement. Hydrogen atoms attached to carbon atoms were placed in calculated positions and refined using a riding model.

## Supplementary Material

Crystal structure: contains datablock(s) I. DOI: 10.1107/S2056989026005116/hb8202sup1.cif

Structure factors: contains datablock(s) I. DOI: 10.1107/S2056989026005116/hb8202Isup3.hkl

CCDC reference: 2554327

Additional supporting information:  crystallographic information; 3D view; checkCIF report

Additional supporting information:  crystallographic information; 3D view; checkCIF report

## Figures and Tables

**Table 1:** Selected geometric parameters (Å, °)

Cu1—O5*B*	1.953 (3)	Cu2—O6*B*	1.944 (3)
Cu1—O4*A*	1.962 (3)	Cu2—O4	1.972 (3)
Cu1—N3*A*	1.999 (4)	Cu2—N2	2.013 (4)
Cu1—N2*A*	2.009 (4)	Cu2—N3	2.014 (4)
Cu1—O4	2.314 (3)	Cu2—O4*A*	2.484 (3)
			
O5*B*—Cu1—O4*A*	95.02 (12)	N3*A*—Cu1—O4	111.85 (15)
O5*B*—Cu1—N3*A*	90.89 (14)	N2*A*—Cu1—O4	85.08 (14)
O4*A*—Cu1—N3*A*	163.85 (16)	O6*B*—Cu2—O4	91.81 (12)
O5*B*—Cu1—N2*A*	169.40 (14)	O6*B*—Cu2—N2	89.88 (14)
O4*A*—Cu1—N2*A*	94.28 (15)	O4—Cu2—N2	175.61 (14)
N3*A*—Cu1—N2*A*	81.49 (16)	O6*B*—Cu2—N3	169.52 (15)
O5*B*—Cu1—O4	90.99 (12)	O4—Cu2—N3	96.28 (14)
O4*A*—Cu1—O4	83.11 (12)	N2—Cu2—N3	81.59 (16)

**Table 2:** Hydrogen-bond geometry (Å, °)

*D*—H⋯*A*	*D*—H	H⋯*A*	*D*⋯*A*	*D*—H⋯*A*
O6—H6⋯O20*W*	0.82	1.84	2.649 (7)	168
C9—H9⋯O6*B*	0.93	2.48	2.972 (6)	113
C9*A*—H9*A*⋯O1*A*	0.93	2.52	3.217 (7)	132
C18—H18⋯O5	0.93	2.24	3.150 (7)	166
C18*A*—H18*A*⋯O5*B*	0.93	2.55	3.017 (6)	112
C18*A*—H18*A*⋯O3*B*	0.93	2.46	3.365 (7)	165
C6—H6*C*⋯O3*B*^i^	0.93	2.58	3.252 (7)	129
C17*A*—H17*A*⋯O2^i^	0.93	2.11	3.027 (8)	169
O4*B*—H4*B*⋯O5*A*	0.88 (6)	1.70 (6)	2.512 (7)	151 (6)

Symmetry code: (i) 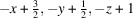.

**Table 3:** Experimental details

Crystal data	
Chemical formula	[Cu_4_(C_8_H_4_NO_6_)_4_(C_8_H_3_NO_6_)_2_(C_12_H_8_N_2_)_4_]·6H_2_O
*M*_r_	2329.69
Crystal system, space group	Monoclinic, *C*2/*c*
Temperature (K)	293
*a*, *b*, *c* (Å)	24.890 (3), 14.3705 (7), 30.179 (3)
β (°)	116.919 (12)
*V* (Å^3^)	9624.8 (17)
*Z*	4
Radiation type	Cu *K*α
μ (mm^−1^)	1.90
Crystal size (mm)	0.04 × 0.03 × 0.01

Data collection	
Diffractometer	Xcalibur, Ruby
Absorption correction	Multi-scan (*CrysAlis PRO*; Agilent Technologies, 2014 ▸)
*T*_min_, *T*_max_	0.916, 1.000
No. of measured, independent and observed [*I* > 2σ(*I*)] reflections	33685, 9915, 5785
*R*_int_	0.057
(sin θ/λ)_max_ (Å^−1^)	0.632

Refinement	
*R*[*F*^2^ > 2σ(*F*^2^)], *wR*(*F*^2^), *S*	0.061, 0.181, 1.01
No. of reflections	9915
No. of parameters	707
H-atom treatment	H atoms treated by a mixture of independent and constrained refinement
Δρ_max_, Δρ_min_ (e Å^−3^)	0.29, −0.30

Computer programs: *CrysAlis PRO* (Agilent, 2014 ▸), *SHELXT* (Sheldrick, 2015*a* ▸) and *SHELXL2025/1* (Sheldrick, 2015*b* ▸).

## supplementary crystallographic information

### Tetrakis(µ-2-carboxy-6-nitrobenzoato-κ2O:O')bis(µ-3-nitrobenzene-1,2-dicarboxylato-κ2O:O')tetrakis[(1,10-phenanthroline-κN:N')copper(II)] hexahydrate Crystal data

**Table:**

[Cu_4_(C_8_H_4_NO_6_)_4_(C_8_H_3_NO_6_)_2_(C_12_H_8_N_2_)_4_]·6H_2_O	*F*(000) = 4720
*M**_r_* = 2329.69	*D*_x_ = 1.608 Mg m^−^^3^
Monoclinic, *C*2/*c*	Cu *K*α radiation, λ = 1.54184 Å
*a* = 24.890 (3) Å	Cell parameters from 4168 reflections
*b* = 14.3705 (7) Å	θ = 3.6–60.4°
*c* = 30.179 (3) Å	µ = 1.90 mm^−^^1^
β = 116.919 (12)°	*T* = 293 K
*V* = 9624.8 (17) Å^3^	Prism, blue
*Z* = 4	0.04 × 0.03 × 0.01 mm

### Tetrakis(µ-2-carboxy-6-nitrobenzoato-κ2O:O')bis(µ-3-nitrobenzene-1,2-dicarboxylato-κ2O:O')tetrakis[(1,10-phenanthroline-κN:N')copper(II)] hexahydrate Data collection

**Table:**

Xcalibur, Ruby diffractometer	*R*_int_ = 0.057
ω scans	θ_max_ = 77.0°, θ_min_ = 3.3°
Absorption correction: multi-scan (CrysAlisPro; Agilent Technologies, 2014)	*h* = −29→31
*T*_min_ = 0.916, *T*_max_ = 1.000	*k* = −14→17
33685 measured reflections	*l* = −37→37
9915 independent reflections	3 standard reflections every 100 reflections
5785 reflections with *I* > 2σ(*I*)	intensity decay: 2.6%

### Tetrakis(µ-2-carboxy-6-nitrobenzoato-κ2O:O')bis(µ-3-nitrobenzene-1,2-dicarboxylato-κ2O:O')tetrakis[(1,10-phenanthroline-κN:N')copper(II)] hexahydrate Refinement

**Table:**

Refinement on *F*^2^	Primary atom site location: dual
Least-squares matrix: full	Hydrogen site location: mixed
*R*[*F*^2^ > 2σ(*F*^2^)] = 0.061	H atoms treated by a mixture of independent and constrained refinement
*wR*(*F*^2^) = 0.181	*w* = 1/[σ^2^(*F*_o_^2^) + (0.078*P*)^2^ + 6.3715*P*] where *P* = (*F*_o_^2^ + 2*F*_c_^2^)/3
*S* = 1.01	(Δ/σ)_max_ = 0.001
9915 reflections	Δρ_max_ = 0.29 e Å^−^^3^
707 parameters	Δρ_min_ = −0.30 e Å^−^^3^
0 restraints	

### Tetrakis(µ-2-carboxy-6-nitrobenzoato-κ2O:O')bis(µ-3-nitrobenzene-1,2-dicarboxylato-κ2O:O')tetrakis[(1,10-phenanthroline-κN:N')copper(II)] hexahydrate Special details

**Table:**

Geometry. All esds (except the esd in the dihedral angle between two l.s. planes) are estimated using the full covariance matrix. The cell esds are taken into account individually in the estimation of esds in distances, angles and torsion angles; correlations between esds in cell parameters are only used when they are defined by crystal symmetry. An approximate (isotropic) treatment of cell esds is used for estimating esds involving l.s. planes.

### Tetrakis(µ-2-carboxy-6-nitrobenzoato-κ2O:O')bis(µ-3-nitrobenzene-1,2-dicarboxylato-κ2O:O')tetrakis[(1,10-phenanthroline-κN:N')copper(II)] hexahydrate Fractional atomic coordinates and isotropic or equivalent isotropic displacement parameters (Å^2^)

**Table:**

	*x*	*y*	*z*	*U*_iso_*/*U*_eq_	
Cu1	0.68380 (3)	0.40513 (4)	0.62556 (3)	0.0663 (2)	
Cu2	0.57301 (3)	0.55106 (4)	0.58128 (3)	0.0681 (2)	
O4	0.58184 (14)	0.41696 (19)	0.57240 (11)	0.0638 (7)	
O5B	0.70341 (14)	0.47576 (19)	0.57949 (12)	0.0681 (8)	
O4A	0.66823 (15)	0.5151 (2)	0.65655 (11)	0.0687 (8)	
O6B	0.63082 (14)	0.5829 (2)	0.55696 (12)	0.0683 (8)	
O3	0.49041 (16)	0.3972 (2)	0.50867 (13)	0.0779 (9)	
O3A	0.76759 (18)	0.5158 (2)	0.70616 (15)	0.0917 (11)	
N2	0.56699 (17)	0.6862 (2)	0.59599 (14)	0.0671 (9)	
O5	0.49952 (19)	0.2586 (3)	0.58544 (15)	0.0955 (11)	
N2A	0.65854 (18)	0.3142 (3)	0.66320 (13)	0.0700 (10)	
N3	0.52032 (18)	0.5369 (3)	0.61551 (15)	0.0750 (11)	
O4B	0.8250 (2)	0.5670 (4)	0.60462 (18)	0.1019 (13)	
N3A	0.71965 (19)	0.2900 (3)	0.61278 (15)	0.0741 (10)	
O1	0.5857 (2)	0.4276 (3)	0.47776 (14)	0.0961 (11)	
O5A	0.75695 (19)	0.6524 (3)	0.63184 (16)	0.1052 (13)	
O2B	0.8629 (2)	0.6170 (3)	0.5234 (2)	0.1107 (14)	
O1A	0.6748 (2)	0.5141 (3)	0.75437 (17)	0.1106 (13)	
N1B	0.8179 (3)	0.6167 (3)	0.4837 (2)	0.0887 (14)	
O3B	0.8163 (2)	0.4526 (3)	0.5536 (2)	0.1175 (16)	
O6	0.5031 (2)	0.1049 (3)	0.57957 (19)	0.1154 (15)	
H6	0.487224	0.106557	0.598203	0.173*	
C8B	0.6742 (2)	0.5460 (3)	0.55488 (16)	0.0617 (10)	
N1A	0.6523 (3)	0.5930 (4)	0.74713 (19)	0.1032 (15)	
C1	0.5413 (2)	0.3717 (3)	0.53517 (18)	0.0634 (11)	
C2	0.5618 (2)	0.2773 (3)	0.52519 (17)	0.0654 (11)	
C1A	0.7175 (3)	0.5513 (3)	0.68901 (19)	0.0707 (12)	
C19A	0.7050 (2)	0.2121 (3)	0.63054 (18)	0.0701 (12)	
C20A	0.6711 (2)	0.2256 (3)	0.65673 (16)	0.0687 (12)	
C3B	0.6927 (2)	0.5888 (3)	0.51872 (17)	0.0644 (11)	
C3	0.5471 (2)	0.1928 (3)	0.53959 (18)	0.0709 (12)	
N1	0.6128 (3)	0.3575 (4)	0.4818 (2)	0.1069 (17)	
C7B	0.7586 (2)	0.6211 (3)	0.48319 (19)	0.0703 (12)	
C20	0.5331 (2)	0.7002 (3)	0.62056 (19)	0.0745 (13)	
O1B	0.8192 (3)	0.6149 (4)	0.4437 (2)	0.1395 (19)	
C2B	0.7503 (2)	0.5805 (3)	0.52163 (18)	0.0658 (11)	
C1B	0.8005 (3)	0.5276 (4)	0.5624 (2)	0.0838 (15)	
C12A	0.6527 (3)	0.1489 (3)	0.67555 (18)	0.0811 (15)	
C4B	0.6482 (2)	0.6370 (3)	0.47936 (19)	0.0780 (13)	
H4BA	0.610222	0.643114	0.477832	0.094*	
C7	0.5951 (3)	0.2709 (3)	0.49873 (19)	0.0818 (14)	
C2A	0.7119 (2)	0.6478 (3)	0.70521 (18)	0.0783 (14)	
C8	0.5140 (2)	0.1911 (4)	0.5699 (2)	0.0809 (14)	
C7A	0.6800 (3)	0.6674 (4)	0.7313 (2)	0.0872 (15)	
C9	0.5893 (2)	0.7587 (3)	0.58342 (19)	0.0767 (13)	
H9	0.612557	0.749391	0.566833	0.092*	
C19	0.5086 (2)	0.6199 (4)	0.6313 (2)	0.0795 (14)	
C15A	0.7218 (3)	0.1221 (4)	0.6232 (2)	0.0891 (16)	
C3A	0.7410 (3)	0.7233 (4)	0.69456 (19)	0.0881 (16)	
C6B	0.7145 (3)	0.6669 (3)	0.4439 (2)	0.0874 (16)	
H6B	0.721880	0.691329	0.418653	0.105*	
C9A	0.6260 (2)	0.3303 (4)	0.68692 (19)	0.0827 (14)	
H9A	0.616953	0.391459	0.691125	0.099*	
C18	0.4977 (3)	0.4609 (4)	0.6249 (2)	0.0906 (16)	
H18	0.504946	0.404131	0.613777	0.109*	
C10	0.5788 (3)	0.8502 (4)	0.5945 (2)	0.0933 (17)	
H10	0.594584	0.900715	0.585008	0.112*	
C5B	0.6590 (3)	0.6758 (4)	0.4427 (2)	0.0889 (16)	
H5B	0.628610	0.708421	0.416909	0.107*	
C4	0.5627 (3)	0.1082 (4)	0.5251 (2)	0.0892 (16)	
H4	0.551056	0.052518	0.533840	0.107*	
C13A	0.6694 (3)	0.0585 (4)	0.6670 (2)	0.105 (2)	
H13	0.657078	0.006929	0.678589	0.126*	
C12	0.5212 (3)	0.7884 (4)	0.6328 (2)	0.0962 (18)	
C18A	0.7520 (3)	0.2806 (4)	0.5886 (3)	0.1020 (19)	
H18A	0.762359	0.333169	0.576161	0.122*	
C14A	0.7026 (3)	0.0453 (4)	0.6425 (2)	0.105 (2)	
H14	0.713100	−0.014861	0.638137	0.126*	
O6A	0.8244 (3)	0.7556 (4)	0.67908 (18)	0.176 (3)	
O2A	0.6074 (3)	0.6095 (5)	0.7516 (3)	0.171 (3)	
C10A	0.6049 (3)	0.2582 (5)	0.7059 (2)	0.1007 (19)	
H10A	0.581844	0.271313	0.722211	0.121*	
C6	0.6117 (3)	0.1874 (4)	0.4856 (2)	0.1042 (19)	
H6C	0.634373	0.186840	0.468176	0.125*	
C11A	0.6183 (3)	0.1689 (5)	0.7002 (2)	0.103 (2)	
H11	0.604545	0.120620	0.712941	0.124*	
C8A	0.7766 (3)	0.7104 (4)	0.6670 (2)	0.106 (2)	
C5	0.5944 (3)	0.1058 (4)	0.4986 (2)	0.103 (2)	
H5	0.604318	0.049097	0.489350	0.123*	
C11	0.5460 (3)	0.8637 (4)	0.6189 (3)	0.105 (2)	
H11A	0.539583	0.923959	0.626724	0.126*	
C6A	0.6737 (4)	0.7582 (5)	0.7465 (2)	0.118 (2)	
H6AA	0.650833	0.769487	0.763242	0.141*	
C4A	0.7378 (4)	0.8113 (4)	0.7111 (2)	0.123 (3)	
H4A	0.759020	0.860000	0.706021	0.147*	
O2	0.6580 (4)	0.3537 (4)	0.4755 (3)	0.202 (3)	
C16A	0.7568 (3)	0.1152 (4)	0.5989 (3)	0.118 (2)	
H16	0.770545	0.057285	0.594687	0.141*	
C15	0.4734 (3)	0.6262 (5)	0.6566 (3)	0.109 (2)	
C17	0.4633 (3)	0.4621 (5)	0.6509 (3)	0.118 (2)	
H17	0.448451	0.406855	0.657069	0.142*	
C16	0.4519 (4)	0.5432 (5)	0.6668 (3)	0.132 (3)	
H16A	0.429680	0.544244	0.684613	0.158*	
C13	0.4841 (4)	0.7939 (5)	0.6585 (3)	0.138 (3)	
H13A	0.475532	0.851723	0.667570	0.166*	
C5A	0.7023 (4)	0.8275 (5)	0.7357 (3)	0.129 (3)	
H5A	0.698483	0.888045	0.744745	0.155*	
C17A	0.7712 (4)	0.1920 (5)	0.5811 (3)	0.128 (3)	
H17A	0.793959	0.186844	0.563752	0.154*	
C14	0.4620 (4)	0.7163 (6)	0.6695 (3)	0.143 (3)	
H14A	0.438439	0.722208	0.686080	0.172*	
O20W	0.4518 (2)	0.0830 (4)	0.6390 (2)	0.153 (2)	
O22W	0.4510 (4)	0.2324 (7)	0.6994 (3)	0.235 (4)	
O23W	0.8642 (4)	0.4422 (6)	0.6892 (3)	0.237 (4)	
H4B	0.807 (3)	0.613 (4)	0.612 (2)	0.10 (2)*	

### Tetrakis(µ-2-carboxy-6-nitrobenzoato-κ2O:O')bis(µ-3-nitrobenzene-1,2-dicarboxylato-κ2O:O')tetrakis[(1,10-phenanthroline-κN:N')copper(II)] hexahydrate Atomic displacement parameters (Å^2^)

**Table:**

	*U*^11^	*U*^22^	*U*^33^	*U*^12^	*U*^13^	*U*^23^
Cu1	0.0866 (5)	0.0514 (3)	0.0755 (4)	−0.0006 (3)	0.0495 (4)	0.0031 (3)
Cu2	0.0811 (4)	0.0560 (3)	0.0841 (5)	−0.0046 (3)	0.0524 (4)	−0.0123 (3)
O4	0.0750 (19)	0.0560 (16)	0.0661 (18)	−0.0035 (14)	0.0369 (16)	−0.0106 (14)
O5B	0.084 (2)	0.0547 (16)	0.083 (2)	0.0085 (14)	0.0531 (18)	0.0135 (15)
O4A	0.089 (2)	0.0560 (16)	0.0697 (19)	−0.0092 (15)	0.0436 (18)	−0.0073 (14)
O6B	0.076 (2)	0.0585 (16)	0.085 (2)	0.0074 (14)	0.0493 (18)	0.0046 (15)
O3	0.080 (2)	0.0674 (19)	0.079 (2)	0.0144 (16)	0.0293 (19)	−0.0058 (16)
O3A	0.090 (3)	0.069 (2)	0.103 (3)	0.0090 (19)	0.033 (2)	0.0103 (19)
N2	0.076 (2)	0.055 (2)	0.069 (2)	0.0011 (17)	0.032 (2)	−0.0082 (17)
O5	0.126 (3)	0.070 (2)	0.109 (3)	−0.017 (2)	0.069 (3)	−0.011 (2)
N2A	0.083 (3)	0.067 (2)	0.062 (2)	−0.0137 (19)	0.035 (2)	0.0017 (18)
N3	0.081 (3)	0.077 (3)	0.085 (3)	−0.011 (2)	0.053 (2)	−0.018 (2)
O4B	0.088 (3)	0.124 (4)	0.098 (3)	0.005 (3)	0.047 (3)	0.023 (3)
N3A	0.089 (3)	0.060 (2)	0.084 (3)	0.0082 (19)	0.048 (2)	0.0077 (19)
O1	0.139 (4)	0.076 (2)	0.087 (3)	0.010 (2)	0.063 (3)	0.0061 (19)
O5A	0.111 (3)	0.096 (3)	0.097 (3)	−0.032 (2)	0.037 (2)	−0.013 (2)
O2B	0.111 (3)	0.101 (3)	0.154 (4)	0.000 (3)	0.090 (3)	0.010 (3)
O1A	0.145 (4)	0.103 (3)	0.101 (3)	−0.011 (3)	0.071 (3)	−0.001 (3)
N1B	0.125 (4)	0.056 (2)	0.126 (4)	−0.002 (3)	0.093 (4)	0.004 (3)
O3B	0.131 (4)	0.080 (2)	0.189 (5)	0.038 (2)	0.115 (4)	0.039 (3)
O6	0.113 (3)	0.070 (2)	0.176 (5)	−0.006 (2)	0.077 (3)	0.018 (3)
C8B	0.071 (3)	0.055 (2)	0.068 (3)	−0.001 (2)	0.039 (2)	0.001 (2)
N1A	0.113 (4)	0.121 (4)	0.082 (3)	0.007 (4)	0.050 (3)	−0.016 (3)
C1	0.075 (3)	0.052 (2)	0.071 (3)	0.001 (2)	0.039 (3)	−0.004 (2)
C2	0.071 (3)	0.057 (2)	0.063 (3)	0.005 (2)	0.026 (2)	−0.0066 (19)
C1A	0.091 (4)	0.056 (2)	0.072 (3)	−0.002 (3)	0.042 (3)	0.004 (2)
C19A	0.079 (3)	0.049 (2)	0.068 (3)	0.001 (2)	0.021 (2)	0.005 (2)
C20A	0.081 (3)	0.060 (3)	0.055 (2)	−0.007 (2)	0.022 (2)	0.005 (2)
C3B	0.084 (3)	0.051 (2)	0.068 (3)	0.002 (2)	0.043 (2)	0.003 (2)
C3	0.070 (3)	0.056 (2)	0.074 (3)	0.005 (2)	0.021 (2)	−0.008 (2)
N1	0.162 (5)	0.088 (3)	0.113 (4)	0.012 (3)	0.100 (4)	0.001 (3)
C7B	0.099 (4)	0.053 (2)	0.082 (3)	−0.004 (2)	0.061 (3)	−0.004 (2)
C20	0.075 (3)	0.077 (3)	0.080 (3)	−0.001 (2)	0.043 (3)	−0.019 (2)
O1B	0.196 (5)	0.144 (4)	0.154 (4)	−0.003 (4)	0.146 (4)	−0.001 (3)
C2B	0.088 (3)	0.048 (2)	0.078 (3)	0.003 (2)	0.052 (3)	0.003 (2)
C1B	0.089 (4)	0.084 (4)	0.103 (4)	0.002 (3)	0.065 (4)	0.019 (3)
C12A	0.098 (4)	0.069 (3)	0.056 (3)	−0.022 (3)	0.017 (3)	0.006 (2)
C4B	0.091 (4)	0.067 (3)	0.082 (3)	0.001 (3)	0.045 (3)	0.009 (2)
C7	0.105 (4)	0.069 (3)	0.076 (3)	0.015 (3)	0.045 (3)	−0.004 (2)
C2A	0.096 (4)	0.064 (3)	0.057 (3)	0.004 (3)	0.020 (3)	−0.003 (2)
C8	0.068 (3)	0.063 (3)	0.095 (4)	−0.009 (2)	0.023 (3)	0.003 (3)
C7A	0.098 (4)	0.076 (3)	0.069 (3)	−0.001 (3)	0.021 (3)	−0.008 (3)
C9	0.085 (3)	0.064 (3)	0.084 (3)	0.002 (2)	0.040 (3)	0.001 (2)
C19	0.089 (3)	0.082 (3)	0.083 (3)	−0.011 (3)	0.053 (3)	−0.027 (3)
C15A	0.103 (4)	0.061 (3)	0.084 (4)	0.009 (3)	0.025 (3)	−0.001 (3)
C3A	0.110 (4)	0.067 (3)	0.061 (3)	−0.016 (3)	0.015 (3)	0.001 (2)
C6B	0.134 (5)	0.066 (3)	0.079 (3)	−0.001 (3)	0.063 (4)	0.007 (3)
C9A	0.099 (4)	0.085 (3)	0.078 (3)	−0.013 (3)	0.052 (3)	−0.001 (3)
C18	0.108 (4)	0.084 (4)	0.105 (4)	−0.019 (3)	0.071 (4)	−0.017 (3)
C10	0.109 (4)	0.060 (3)	0.116 (5)	−0.002 (3)	0.056 (4)	−0.006 (3)
C5B	0.110 (4)	0.081 (3)	0.071 (3)	−0.001 (3)	0.037 (3)	0.014 (3)
C4	0.085 (4)	0.063 (3)	0.102 (4)	0.007 (3)	0.027 (3)	−0.006 (3)
C13A	0.140 (6)	0.063 (3)	0.081 (4)	−0.022 (3)	0.023 (4)	0.010 (3)
C12	0.101 (4)	0.079 (4)	0.118 (5)	−0.005 (3)	0.058 (4)	−0.037 (3)
C18A	0.122 (5)	0.078 (3)	0.142 (5)	0.012 (3)	0.090 (5)	0.010 (3)
C14A	0.137 (6)	0.056 (3)	0.094 (4)	0.010 (3)	0.029 (4)	0.009 (3)
O6A	0.225 (6)	0.207 (6)	0.098 (3)	−0.142 (5)	0.074 (4)	−0.034 (4)
O2A	0.159 (5)	0.214 (7)	0.194 (6)	0.015 (5)	0.128 (5)	−0.011 (5)
C10A	0.118 (5)	0.112 (5)	0.084 (4)	−0.035 (4)	0.056 (4)	−0.007 (3)
C6	0.131 (5)	0.091 (4)	0.106 (4)	0.026 (4)	0.067 (4)	−0.016 (3)
C11A	0.122 (5)	0.111 (5)	0.073 (4)	−0.036 (4)	0.041 (4)	0.007 (3)
C8A	0.151 (6)	0.091 (4)	0.068 (3)	−0.041 (4)	0.041 (4)	0.003 (3)
C5	0.118 (5)	0.069 (3)	0.113 (5)	0.021 (3)	0.045 (4)	−0.020 (3)
C11	0.111 (5)	0.072 (4)	0.131 (5)	0.007 (3)	0.054 (4)	−0.026 (4)
C6A	0.145 (6)	0.097 (5)	0.085 (4)	0.023 (4)	0.029 (4)	−0.024 (4)
C4A	0.172 (7)	0.056 (3)	0.081 (4)	−0.002 (4)	0.005 (4)	0.001 (3)
O2	0.304 (9)	0.134 (4)	0.322 (9)	0.032 (5)	0.277 (8)	0.016 (5)
C16A	0.149 (6)	0.073 (4)	0.143 (6)	0.035 (4)	0.077 (5)	0.007 (4)
C15	0.124 (5)	0.115 (5)	0.125 (5)	−0.016 (4)	0.088 (5)	−0.046 (4)
C17	0.138 (6)	0.119 (5)	0.142 (6)	−0.036 (4)	0.103 (5)	−0.026 (5)
C16	0.165 (7)	0.139 (6)	0.157 (7)	−0.046 (5)	0.130 (6)	−0.055 (5)
C13	0.152 (7)	0.116 (6)	0.183 (8)	−0.010 (5)	0.107 (6)	−0.074 (6)
C5A	0.173 (8)	0.067 (4)	0.098 (5)	0.015 (4)	0.017 (5)	−0.022 (4)
C17A	0.155 (7)	0.098 (5)	0.183 (8)	0.023 (4)	0.123 (6)	0.000 (5)
C14	0.170 (8)	0.131 (6)	0.194 (8)	−0.031 (5)	0.140 (7)	−0.066 (6)
O20W	0.104 (4)	0.193 (5)	0.160 (5)	−0.027 (3)	0.057 (3)	0.042 (4)
O22W	0.218 (8)	0.310 (10)	0.242 (8)	−0.020 (7)	0.163 (7)	−0.008 (7)
O23W	0.231 (8)	0.298 (10)	0.239 (8)	0.104 (7)	0.156 (7)	0.163 (7)

### Tetrakis(µ-2-carboxy-6-nitrobenzoato-κ2O:O')bis(µ-3-nitrobenzene-1,2-dicarboxylato-κ2O:O')tetrakis[(1,10-phenanthroline-κN:N')copper(II)] hexahydrate Geometric parameters (Å, º)

**Table:**

Cu1—O5B	1.953 (3)	C12A—C11A	1.396 (8)
Cu1—O4A	1.962 (3)	C12A—C13A	1.423 (8)
Cu1—N3A	1.999 (4)	C4B—C5B	1.370 (7)
Cu1—N2A	2.009 (4)	C4B—H4BA	0.9300
Cu1—O4	2.314 (3)	C7—C6	1.384 (7)
Cu2—O6B	1.944 (3)	C2A—C7A	1.377 (8)
Cu2—O4	1.972 (3)	C2A—C3A	1.419 (7)
Cu2—N2	2.013 (4)	C7A—C6A	1.415 (8)
Cu2—N3	2.014 (4)	C9—C10	1.411 (7)
Cu2—O4A	2.484 (3)	C9—H9	0.9300
O4—C1	1.295 (5)	C19—C15	1.405 (7)
O5B—C8B	1.269 (5)	C15A—C16A	1.375 (9)
O4A—C1A	1.283 (6)	C15A—C14A	1.428 (9)
O6B—C8B	1.229 (5)	C3A—C4A	1.375 (8)
O3—C1	1.210 (5)	C3A—C8A	1.476 (9)
O3A—C1A	1.224 (6)	C6B—C5B	1.370 (8)
N2—C9	1.315 (6)	C6B—H6B	0.9300
N2—C20	1.367 (6)	C9A—C10A	1.396 (7)
O5—C8	1.201 (6)	C9A—H9A	0.9300
N2A—C9A	1.321 (6)	C18—C17	1.399 (8)
N2A—C20A	1.347 (6)	C18—H18	0.9300
N3—C18	1.316 (6)	C10—C11	1.337 (8)
N3—C19	1.364 (6)	C10—H10	0.9300
O4B—C1B	1.269 (7)	C5B—H5B	0.9300
O4B—H4B	0.88 (6)	C4—C5	1.352 (8)
N3A—C18A	1.317 (7)	C4—H4	0.9300
N3A—C19A	1.360 (6)	C13A—C14A	1.345 (9)
O1—N1	1.188 (6)	C13A—H13	0.9300
O5A—C8A	1.261 (7)	C12—C11	1.402 (9)
O2B—N1B	1.216 (7)	C12—C13	1.452 (9)
O1A—N1A	1.240 (6)	C18A—C17A	1.414 (8)
N1B—O1B	1.220 (6)	C18A—H18A	0.9300
N1B—C7B	1.471 (7)	C14A—H14	0.9300
O3B—C1B	1.218 (6)	O6A—C8A	1.256 (8)
O6—C8	1.328 (6)	C10A—C11A	1.356 (9)
O6—H6	0.8200	C10A—H10A	0.9300
C8B—C3B	1.496 (6)	C6—C5	1.367 (9)
N1A—O2A	1.207 (7)	C6—H6C	0.9300
N1A—C7A	1.465 (8)	C11A—H11	0.9300
C1—C2	1.526 (6)	C5—H5	0.9300
C2—C7	1.391 (7)	C11—H11A	0.9300
C2—C3	1.394 (6)	C6A—C5A	1.349 (11)
C1A—C2A	1.499 (7)	C6A—H6AA	0.9300
C19A—C15A	1.407 (7)	C4A—C5A	1.406 (11)
C19A—C20A	1.405 (7)	C4A—H4A	0.9300
C20A—C12A	1.408 (6)	C16A—C17A	1.347 (9)
C3B—C4B	1.388 (7)	C16A—H16	0.9300
C3B—C2B	1.401 (6)	C15—C16	1.396 (9)
C3—C4	1.406 (7)	C15—C14	1.417 (9)
C3—C8	1.483 (8)	C17—C16	1.339 (9)
N1—O2	1.225 (7)	C17—H17	0.9300
N1—C7	1.485 (7)	C16—H16A	0.9300
C7B—C6B	1.366 (7)	C13—C14	1.350 (10)
C7B—C2B	1.395 (6)	C13—H13A	0.9300
C20—C12	1.388 (7)	C5A—H5A	0.9300
C20—C19	1.409 (7)	C17A—H17A	0.9300
C2B—C1B	1.504 (7)	C14—H14A	0.9300
			
O5B—Cu1—O4A	95.02 (12)	O6—C8—C3	112.1 (5)
O5B—Cu1—N3A	90.89 (14)	C2A—C7A—C6A	123.5 (6)
O4A—Cu1—N3A	163.85 (16)	C2A—C7A—N1A	121.1 (5)
O5B—Cu1—N2A	169.40 (14)	C6A—C7A—N1A	115.4 (6)
O4A—Cu1—N2A	94.28 (15)	N2—C9—C10	121.5 (5)
N3A—Cu1—N2A	81.49 (16)	N2—C9—H9	119.3
O5B—Cu1—O4	90.99 (12)	C10—C9—H9	119.3
O4A—Cu1—O4	83.11 (12)	N3—C19—C15	122.1 (5)
N3A—Cu1—O4	111.85 (15)	N3—C19—C20	117.0 (4)
N2A—Cu1—O4	85.08 (14)	C15—C19—C20	120.9 (5)
O6B—Cu2—O4	91.81 (12)	C16A—C15A—C19A	117.1 (5)
O6B—Cu2—N2	89.88 (14)	C16A—C15A—C14A	125.0 (6)
O4—Cu2—N2	175.61 (14)	C19A—C15A—C14A	117.9 (6)
O6B—Cu2—N3	169.52 (15)	C4A—C3A—C2A	120.1 (7)
O4—Cu2—N3	96.28 (14)	C4A—C3A—C8A	117.9 (6)
N2—Cu2—N3	81.59 (16)	C2A—C3A—C8A	122.0 (5)
C1—O4—Cu2	121.2 (3)	C7B—C6B—C5B	118.2 (5)
C1—O4—Cu1	138.0 (3)	C7B—C6B—H6B	120.9
Cu2—O4—Cu1	97.60 (12)	C5B—C6B—H6B	120.9
C8B—O5B—Cu1	124.6 (3)	N2A—C9A—C10A	121.9 (5)
C1A—O4A—Cu1	111.3 (3)	N2A—C9A—H9A	119.1
C8B—O6B—Cu2	137.3 (3)	C10A—C9A—H9A	119.0
C9—N2—C20	119.0 (4)	N3—C18—C17	122.7 (5)
C9—N2—Cu2	128.1 (3)	N3—C18—H18	118.7
C20—N2—Cu2	112.8 (3)	C17—C18—H18	118.7
C9A—N2A—C20A	118.9 (4)	C11—C10—C9	119.4 (6)
C9A—N2A—Cu1	128.1 (4)	C11—C10—H10	120.3
C20A—N2A—Cu1	112.3 (3)	C9—C10—H10	120.3
C18—N3—C19	118.1 (4)	C4B—C5B—C6B	120.3 (5)
C18—N3—Cu2	129.4 (3)	C4B—C5B—H5B	119.9
C19—N3—Cu2	112.4 (3)	C6B—C5B—H5B	119.9
C1B—O4B—H4B	121 (4)	C5—C4—C3	121.6 (6)
C18A—N3A—C19A	118.4 (4)	C5—C4—H4	119.2
C18A—N3A—Cu1	129.1 (4)	C3—C4—H4	119.2
C19A—N3A—Cu1	112.5 (3)	C14A—C13A—C12A	122.1 (6)
O2B—N1B—O1B	123.4 (6)	C14A—C13A—H13	119.0
O2B—N1B—C7B	118.8 (5)	C12A—C13A—H13	119.0
O1B—N1B—C7B	117.8 (6)	C20—C12—C11	116.7 (5)
C8—O6—H6	109.5	C20—C12—C13	117.1 (6)
O6B—C8B—O5B	126.7 (4)	C11—C12—C13	126.2 (6)
O6B—C8B—C3B	115.3 (4)	N3A—C18A—C17A	121.2 (6)
O5B—C8B—C3B	118.0 (4)	N3A—C18A—H18A	119.4
O2A—N1A—O1A	121.8 (7)	C17A—C18A—H18A	119.4
O2A—N1A—C7A	119.3 (6)	C13A—C14A—C15A	121.1 (6)
O1A—N1A—C7A	118.9 (5)	C13A—C14A—H14	119.5
O3—C1—O4	126.3 (4)	C15A—C14A—H14	119.5
O3—C1—C2	118.8 (4)	C11A—C10A—C9A	119.4 (6)
O4—C1—C2	114.8 (4)	C11A—C10A—H10A	120.3
C7—C2—C3	115.5 (4)	C9A—C10A—H10A	120.3
C7—C2—C1	120.7 (4)	C5—C6—C7	119.3 (6)
C3—C2—C1	123.7 (4)	C5—C6—H6C	120.4
O3A—C1A—O4A	126.4 (5)	C7—C6—H6C	120.4
O3A—C1A—C2A	118.4 (5)	C10A—C11A—C12A	120.5 (5)
O4A—C1A—C2A	115.2 (5)	C10A—C11A—H11	119.8
N3A—C19A—C15A	122.9 (5)	C12A—C11A—H11	119.8
N3A—C19A—C20A	116.4 (4)	O6A—C8A—O5A	122.8 (7)
C15A—C19A—C20A	120.7 (5)	O6A—C8A—C3A	120.2 (6)
N2A—C20A—C19A	116.7 (4)	O5A—C8A—C3A	117.0 (6)
N2A—C20A—C12A	122.8 (5)	C4—C5—C6	119.4 (5)
C19A—C20A—C12A	120.4 (5)	C4—C5—H5	120.3
C4B—C3B—C2B	119.5 (4)	C6—C5—H5	120.3
C4B—C3B—C8B	116.1 (4)	C10—C11—C12	120.9 (5)
C2B—C3B—C8B	124.3 (4)	C10—C11—H11A	119.5
C2—C3—C4	120.5 (5)	C12—C11—H11A	119.5
C2—C3—C8	120.3 (4)	C5A—C6A—C7A	117.0 (7)
C4—C3—C8	119.3 (5)	C5A—C6A—H6AA	121.5
O1—N1—O2	122.7 (6)	C7A—C6A—H6AA	121.5
O1—N1—C7	120.2 (5)	C3A—C4A—C5A	119.8 (7)
O2—N1—C7	117.0 (5)	C3A—C4A—H4A	120.1
C6B—C7B—C2B	124.1 (5)	C5A—C4A—H4A	120.1
C6B—C7B—N1B	116.0 (5)	C17A—C16A—C15A	120.2 (6)
C2B—C7B—N1B	120.0 (5)	C17A—C16A—H16	119.9
N2—C20—C12	122.5 (5)	C15A—C16A—H16	119.9
N2—C20—C19	116.1 (4)	C16—C15—C19	117.4 (5)
C12—C20—C19	121.4 (5)	C16—C15—C14	125.2 (6)
C7B—C2B—C3B	116.4 (4)	C19—C15—C14	117.4 (6)
C7B—C2B—C1B	120.8 (4)	C16—C17—C18	119.7 (6)
C3B—C2B—C1B	122.7 (4)	C16—C17—H17	120.2
O3B—C1B—O4B	124.0 (6)	C18—C17—H17	120.2
O3B—C1B—C2B	119.9 (6)	C17—C16—C15	120.0 (6)
O4B—C1B—C2B	116.1 (5)	C17—C16—H16A	120.0
C11A—C12A—C20A	116.4 (5)	C15—C16—H16A	120.0
C11A—C12A—C13A	125.9 (6)	C14—C13—C12	121.1 (6)
C20A—C12A—C13A	117.8 (6)	C14—C13—H13A	119.5
C5B—C4B—C3B	121.5 (5)	C12—C13—H13A	119.5
C5B—C4B—H4BA	119.3	C6A—C5A—C4A	122.2 (6)
C3B—C4B—H4BA	119.3	C6A—C5A—H5A	118.9
C6—C7—C2	123.7 (5)	C4A—C5A—H5A	118.9
C6—C7—N1	117.0 (5)	C16A—C17A—C18A	120.2 (6)
C2—C7—N1	119.3 (4)	C16A—C17A—H17A	119.9
C7A—C2A—C3A	117.3 (5)	C18A—C17A—H17A	119.9
C7A—C2A—C1A	122.6 (5)	C13—C14—C15	122.1 (6)
C3A—C2A—C1A	120.1 (5)	C13—C14—H14A	118.9
O5—C8—O6	122.7 (6)	C15—C14—H14A	118.9
O5—C8—C3	125.1 (5)		

### Tetrakis(µ-2-carboxy-6-nitrobenzoato-κ2O:O')bis(µ-3-nitrobenzene-1,2-dicarboxylato-κ2O:O')tetrakis[(1,10-phenanthroline-κN:N')copper(II)] hexahydrate Hydrogen-bond geometry (Å, º)

**Table:**

*D*—H···*A*	*D*—H	H···*A*	*D*···*A*	*D*—H···*A*
O6—H6···O20*W*	0.82	1.84	2.649 (7)	168
C9—H9···O6*B*	0.93	2.48	2.972 (6)	113
C9*A*—H9*A*···O1*A*	0.93	2.52	3.217 (7)	132
C18—H18···O5	0.93	2.24	3.150 (7)	166
C18*A*—H18*A*···O5*B*	0.93	2.55	3.017 (6)	112
C18*A*—H18*A*···O3*B*	0.93	2.46	3.365 (7)	165
C6—H6*C*···O3*B*^i^	0.93	2.58	3.252 (7)	129
C17*A*—H17*A*···O2^i^	0.93	2.11	3.027 (8)	169
O4*B*—H4*B*···O5*A*	0.88 (6)	1.70 (6)	2.512 (7)	151 (6)

Symmetry code: (i) −*x*+3/2, −*y*+1/2, −*z*+1.
